# Donor Site Morbidity and Quality of Life after Microvascular Head and Neck Reconstruction with a Chimeric, Thoracodorsal, Perforator-Scapular Flap Based on the Angular Artery (TDAP-Scap-aa Flap)

**DOI:** 10.3390/jcm11164876

**Published:** 2022-08-19

**Authors:** Jürgen Wallner, Marcus Rieder, Michael Schwaiger, Bernhard Remschmidt, Wolfgang Zemann, Mauro Pau

**Affiliations:** Department of Maxillofacial Surgery, Medical University Graz, 8036 Graz, Austria

**Keywords:** quality-of-life, donor site morbidity, head and neck reconstruction, scapular free flap, oncological outcome, microvascular reconstruction, SF 36, DASH

## Abstract

Extensive defects in the head and neck area often require the use of advanced free flap reconstruction techniques. In this study, the thoracodorsal perforator-scapular free flap technique based on the angular artery (TDAP-Scap-aa flap) was postoperatively evaluated regarding the quality of life and the donor site morbidity using the standardized SF-36 and DASH questionnaires (short form health 36 and disabilities of the arm, shoulder and hand scores). Over a five-year period (2016–2020), 20 selected cases (*n* = 20) requiring both soft and hard tissue reconstruction were assessed. On average, the harvested microvascular free flaps consisted of 7.8 ± 2.1 cm hard tissue and 86 ± 49.8 cm^2^ soft tissue components. At the donor site (subscapular region), only a mild morbidity was observed (DASH score: 21.74 ± 7.3 points). When comparing the patients’ postoperative quality of life to the established values of the healthy German norm population, the observed SF-36 values were within the upper third (>66%) of these established norm values in almost all quality-of-life subcategories. The mild donor site morbidity and the observed quality of life indicate only a small postoperative impairment when using the TDAP-Scap-aa free flap for the reconstruction of extensive maxillofacial defects.

## 1. Introduction

In head and neck surgery, the reconstruction of three-dimensional defects after tumour removal, trauma or osteoradionecrosis poses several technical challenges because both soft and hard tissue components are frequently part of the ablation [1]. In such cases, microvascular free flap techniques are the gold standard for immediate reconstruction of complex maxillomandibular defects [1,2]. The selection of the free flap used depends on varying specific clinical parameters including the size of the defect, the patient’s health status, the prognosis, the surgeon’s preference, the donor site morbidity and the postoperative quality of life [3,4]. The quality of life and the donor site morbidity are of growing importance in reconstructive surgery but particularly in oromandibular defect reconstruction due to the important aesthetical and functional aspects linked to this highly sensitive anatomical area [5].

Although the fibula and the iliac crest have been the most utilized donor sites in the last decades for oromandibular defect reconstruction, the utilization of the subscapular system as an alternative donor site has increased significantly since the year 2000 [6,7,8]. The subscapular system offers the widest array of both hard and soft tissue components that can be used to reconstruct highly complex head and neck defects [4]. Over five dozen permutations of free flaps, based on the subscapular system, are possible [9]. One of these flap configurations is the thoracodorsal, perforator-scapular microvascular free flap based on the angular artery (TDAP-Scap-aa). This technique was first described with a case series of five patients by Pau et al., in 2019 and is particularly suitable for the reconstruction of large defects in the head and neck region [10]. The aim of this study was to assess the donor site morbidity and the postoperative quality of life in patients that underwent extensive maxillomandibular defect reconstruction with the TDAP-Scap-aa free flap technique.

## 2. Materials and Methods

This study was carried out following the local legal requirements and the Declaration of Helsinki (1975) and included the approval of the Ethics Committee of the Medical University of Graz (EK No.: 31-355 ex 18/19). Informed consent was obtained from all subjects involved in the study, and all patients included were treated at the department of Oral and Maxillofacial Surgery at the Medical University Graz, Austria between February 2016 and December 2020.

All patients underwent extensive oromandibular resections including soft and hard tissues followed by simultaneous microvascular reconstruction with a TDAP-Scap-aa free flap. If both sides (left or right) were eligible for flap harvesting (no medical contraindications), reasons for the choice of the harvesting site were the patient’s and the surgeon’s preference.

**Figure 1:**
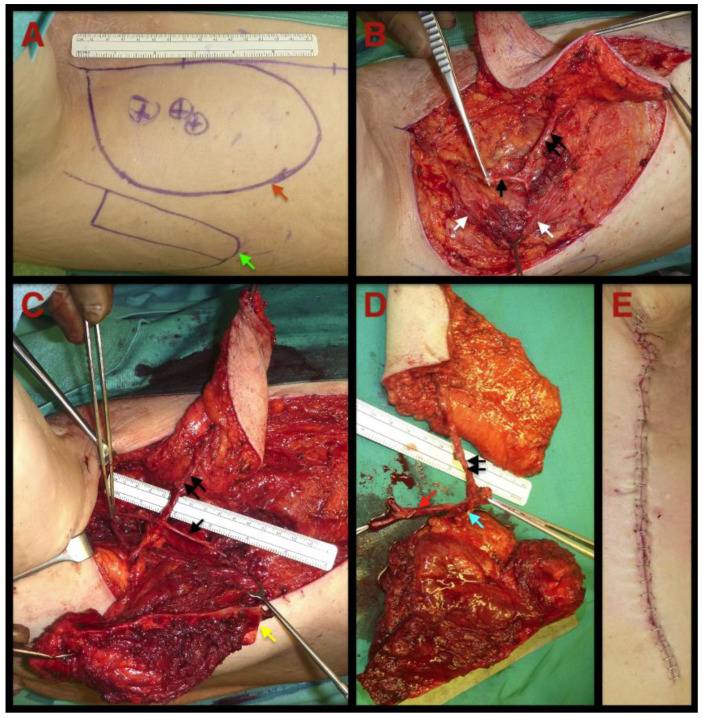
Intraoperative pictures of a 74-year-old male patient while TDAP-Scap-aa flap harvesting: (**A**) flap design: In a lateral decubitus position (arm fixed in 90° abduction) the skin paddle (single orange arrow) and the lateral scapular boarder (single green arrow) are marked. Further, the perforators supplying the skin are marked on the skin paddle after identification with a Doppler ultrasound; (**B**) skin paddle: A pliable muscle-free soft tissue component based on a perforator of the thoracodorsal artery (black double arrow) is dissected after preserving the thoracodorsal nerve (black single arrow) and the latissimus muscle (white single arrow). The skin paddle’s thickness is about 1 cm; (**C**) bone: The lateral scapula boarder is osteomised (hard tissue component—yellow arrow). A muscle cuff is left on the scapula. The harvested bone is supplied by the angular artery. The single black arrow indicates the preserved thoracodorsal nerve. The double black arrow marks the perforator to the skin; (**D**) raised free flap: Soft and hard tissue components are harvested separately. The harvested microvascular bone and the skin paddle can be moved independently from each other. The lateral scapula bone supplied by the angular artery (single blue arrow) and the perforator-based (double black arrow) skin component are both based on the thoracodorsal artery (single red arrow); (**E**) direct wound closure: On the donor site, a primary wound closure can be achieved although large amounts of soft and hard tissues were harvested. Note: Pictures are taken from case number 19.

Figure 1 gives an overview of the flap harvesting procedure. 

The main indications for operation were head and neck squamous cell carcinoma (HNSCC) or osteoradionecrosis (ORN). Over a five-year period (2016–2020), patients fulfilling the following criteria were included in this study: age between 18 and 99 years, complete diagnosis and treatment performed at the abovementioned department, unrestricted preoperative shoulder mobility, ablation and reconstruction procedure in one operation and completed wound healing after operation.

Exclusion criteria were previous operation in the head and neck region, consecutive operation after the primarily curative operation (HNSCC), occurrence of a concomitant disease with hospitalisation after the operation, missing informed consent, lost to follow- up, primary tumour size stages smaller than pT2 (HNSCC) and defect sizes not needing microvascular free flap reconstruction (ORN).

The selected study cohort was investigated retrospectively regarding donor site morbidity and quality of life using the well-established DASH (disabilities of the arm, shoulder and hand) and SF-36 (short form health 36) questionnaires (analysis between 10 and 14 months after surgery). The SF-36 considers eight fields of global health, focusing on physical and emotional aspects, with scores ranging from 0 (poorest health) to 100 (optimal health) [11]. The DASH questionnaire evaluates the overall function of the upper limb. The possible score ranges from 0 (no disability) to 100 (most severe disability) [12,13]. Both questionnaires were chosen with consideration of the availability of the questionnaire in the native language of patients and its worldwide validation. Furthermore, an electronic clinical chart review of the included patients was conducted to collect the demographic patient data including gender, age and TNM-staging (tumour, lymph node metastasis, distant metastasis). Case-specific surgical parameters including defect classification, size and type of flap components and the type of the recipient vessel used for the microvascular anastomosis were collected intraoperatively. Defects of the maxilla were categorized using Brown’s classification of maxillectomy and midface defects (maxillary defect group). Defects of the mandibular were categorized using Cordeiro’s mandibular defect classification (mandibular defect group).

Scores for the SF-36 were automatically calculated using an online open-source tool (OrthoToolKit: https://orthotoolkit.com accessed on 5 June 2021). The DASH questionnaire was evaluated according to its standard protocol. Descriptive and analytical statistics were used to analyse the parameters of this study. The SF-36 scores of the patients who underwent reconstruction with the TDAP-Scap-aa free flap were compared with the already existing data of the healthy German norm population (SF-36 control group) [14]. The DASH scores of the examined patients were compared with already existing values of the United States norm population (DASH control group) [15]. With the aim of determining possible significant differences, the significance (*p*) was calculated with a t-test. For all calculations, a *p*-value of <0,05 was considered as statistically significant. All statistical analyses were performed using the statistical software package SPSS software (IBM Corp. Released 2016. IBM SPSS Statistics for Windows, Version 24.0. Armonk, NY, USA). 

## 3. Results

A total of 20 (*n* = 20) patients was included in this study (mean age 60.0 ± 11.4 years). Thereof, 14 patients (70%) were diagnosed with an HNSCC and 6 patients (30%) with an ORN. The average size of the harvested soft tissue component (skin paddle) was 86 cm^2^ (±49.8; range 16–200 cm^2^). The length of the harvested bone graft ranged from 4 to 12 cm (mean 7.8 ± 2.1 cm) and the width from 2.4 to 3 cm (mean 2.7 ± 0.2 cm). Primary wound closure of the harvesting site could be accomplished in all 20 cases. In 13 cases (65%), the TDAP-Scap-aa free flap was harvested from the right side. In the remaining seven cases (35%), it was harvested from the left side. In all cases, the microvascular anastomosis was performed extra orally in the neck region. The recipient arteries were the facial artery in eight (40%), followed by the superior thyroid artery in seven (35%), the lingual arteries in three (15%) and the external carotid artery in two patients (10%). In one case (5%), the ipsilateral recipient site vessels were not available. Therefore, the contralateral neck was utilised as a source for the recipient vessels. The thoracodorsal nerve was preserved in all 20 cases while harvesting the flap. There were no flap failures observed. All patients received tracheostomy perioperatively. 

### 3.1. Donor Site Morbidity

In the study collective, the DASH score attained a mean score of 21.74 points (± 7.30, range 9.2–35.8) and showed a statistically significant difference (*p* < 0.05) when compared to the mean DASH score of the healthy U.S. population (healthy control group, mean value 10.1 ± 14.68 points). Detailed case, defect and flap characteristics for each patient are shown in Table 1 which also includes the individual postoperative DASH score.

### 3.2. Quality of Life

**Figure 2:**
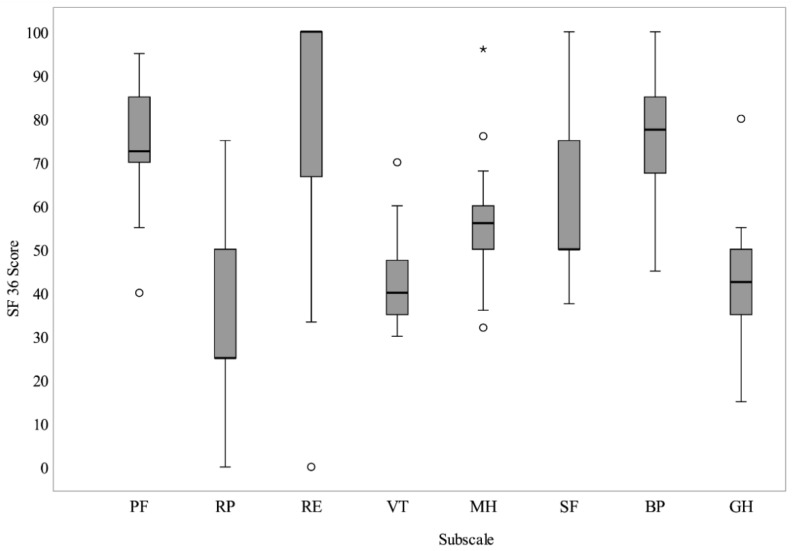
SF-36 results showing the quality-of-life outcome approximately one year (10–14 months) after reconstruction with the TDAP-Scap-aa free flap. Subscales are Physical Functioning (PF), Role Physical (RP), Role Emotional (RE), Vitality (VT), Mental Health (MH), Social Functioning (SF), Bodily Pain (BP) and General Health (GH). * = extreme outlier; ° = mild outlier.

The quality-of-life results evaluated using the SF-36 questionnaire showed several statistically significant differences between the study cohort and the general German population (healthy control group) regarding the subscales vitality, physical functioning, general health perceptions, physical role functioning, social role functioning and mental health (Figure 2). Concerning the subscale emotional role functioning and bodily pain, a one-sample t-test analysis demonstrated that these factors did not differ significantly between the study cohort and the healthy German norm population (healthy control group). Regarding the subscales physical functioning, vitality and mental health, these scores showed an average deterioration of not more than 20 points compared to the German norm population. The largest differences between the results of this study and the healthy control group were found in the physical role (average decline 50 points), the social functioning (average decline 28 points) and the general health subscales (average decline 25 points). The summary data for each subscale and the life quality mean values of the healthy German norm population are shown in Table 2 and Figure 2.

## 4. Discussion

In head and neck surgery, the reconstruction of complex three-dimensional defects with microvascular free flaps is a well-established standard treatment option that should be beneficial for the patient in terms of offering functional and aesthetic enhancement. Therefore, the donor site choice is important for the postoperative outcome and not only depends on the defect size and its anatomical region but also on individual patient factors. The purpose of this study was to present a series of 20 large oromandibular defects including soft and hard tissue which were reconstructed by the TDAP-Scap-aa flap technique [10]. Regarding this microvascular reconstruction method, this study placed particular emphasis on the patients’ postoperative quality of life and the donor site morbidity. 

### 4.1. The TDAP-Scap-aa Flap 

**Figure 3:**
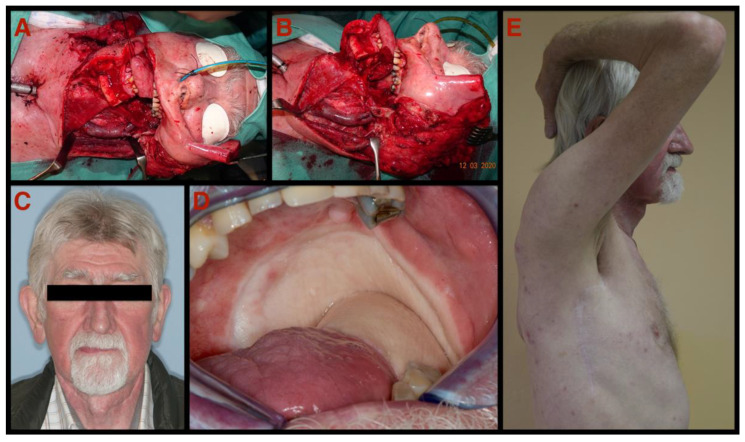
Seventy-four-year-old male patient with squamous cell carcinoma of the oral cavity: (**A**,**B**) intraoperative view after ablation of the squamous cell carcinoma of the oral cavity and modified radical neck dissection; (**C**) postoperative frontal view 8 months after reconstruction with the TDAP-Scap-aa flap; (**D**) intraoral postoperative clinical view depicting the totally integrated transplant involving the mandible, the tongue and the pharyngeal wall 8 months after reconstruction with this microvascular free flap (TDAP-Scap-aa free flap); (**E**) donor site 14 months after surgery. Full range of shoulder movement without restrictions. Note: Pictures are taken from case number 19.

As shown within the data of this study, mainly patients with advanced soft and hard tissue defects (e.g., resulting from T3 or T4 tumour removals) were included in the study cohort. In our series, the size of the harvested skin graft averaged 86 ± 49.8 cm^2^, with the largest skin area measuring up to 200 cm^2^ which allowed a sufficient soft tissue lining of all defects. Since the skin is harvested without the underlying muscle when using the TDAP-scap-aa technique, a comfortable structure lining owing to the reduced soft tissue thickness and volume is possible. This is particularly important if the tongue, the pharynx and/or the palate are part of the soft tissue reconstruction (Figure 3). Consistent with our findings, several research groups reported similar quantities of harvested flap components and described this as one of the eminent benefits of free flaps raised from the subscapular region [10,18,19]. Regarding the bony part of the flap, the length of harvested bone was between 4 and 12 cm (mean 7.8 ± 2.1 cm) long. This hard tissue component allows for the reconstruction of Class III and incomplete Class IV mandibular defects (Brown’s classification of mandibular defects) and large three-dimensional defects of the maxilla [20].

### 4.2. Donor Site Morbidity

Although several studies already describe the donor site morbidity after scapular free flap harvesting to be less severe in comparison to other free flaps, only a few reports assessed the upper limb morbidity with an objective tool [6,8,19,21,22,23,24,25]. Due to this limitation, several research groups stated that further investigations regarding an objective evaluation of postoperative donor site morbidity after scapular free flap harvesting should be conducted [2,19,22]. In 2021, Ferri et al., underpinned the previous assumptions of low donor site morbidity by presenting a series of 19 patients [26].

So far, the present work presents the largest investigation of long-term donor-site morbidity after harvesting a chimeric scapular free flap (TDAP-Scap-aa free flap). Further, similar DASH scores were obtained by a recent study by Janik et al., investigating the serratus anterior free flap (SAFF). Although the SAFF is of course another flap than the TDAP-Scap-aa, it nevertheless belongs to the subscapular artery system or at least one of it branches. Since only soft tissue defects were reconstructed by Janik et al., a microvascular bone component was not part of the harvesting procedure. This indicates that the additional bone harvest from the lateral scapular border seem not to increase donor site morbidity with great significance [27]. 

The present study found a mean DASH score of 21.74 ± 7.3 after investigating 20 patients between 10 to 14 months postoperatively. According to the investigation of Kennedy et al., the obtained DASH score of the present study indicates that patients are able to do their routine work one year after surgery without limitation. Furthermore, the upper-limb disability of the study population can be described as very mild to mild in the five-tier category rating of the donor site morbidity (very mild to very severe) [13]. Although the mean DASH score obtained in this study was found to be statistically higher than the mean DASH score of the healthy U.S. general population (*p* = 0.001), a mild donor site morbidity would support the already published assertion regarding the scapular region’s minimal donor site morbidity [27]. Furthermore, when comparing postoperative morbidity at the donor site with a healthy control group, such as the U.S. population, the impact of a severe diseases on the upper extremity function must be considered, especially when HNSCC or ORN result in an extensive surgical procedure. In addition to that, 75% of the study collective underwent neck dissections which certainly could also have negatively influenced the DASH score [28].

The data of the abovementioned studies conclude that the donor-site morbidity after scapular free flap harvesting is low [6,26,29]. This is in accordance with the findings of this investigation that even after extensive soft and hard tissue harvesting with the TDAP-Scap-aa free flap technique, the donor site morbidity can be described as mild. Furthermore, although high amounts of both soft and hard tissue were harvested in all cases, patients were able to perform their routine work without restrictions one year after surgery.

### 4.3. Quality of Life

In this study, the statistical calculation showed a significant deviation between the study’s quality-of-life results and the healthy German norm population in all subscales except in the subscales bodily pain (*p* = 0.188) and emotional role (*p* = 0.102). Similar observations about the impact of malignancy on health-related quality of life have already been well described in the literature, illustrating that both the primary tumour and the reconstructive surgery negatively influence postoperative quality of life. More precisely, if the primary tumour size increases, the patients’ quality of life deteriorates [30,31,32,33]. Most of the study’s HNSCC patients presented themselves with an advanced tumour stage (T3 and T4 tumours) and therefore required extensive ablative and reconstructive surgery. Furthermore, not only the different types of therapy depending on the tumour stages but also the individual psychological aspects, the proper wound care, the socioeconomic status and the oral hygiene additionally influence the postoperative quality of life [34,35].

The quality-of-life subscale physical role was found to be by far the study’s lowest value scoring roughly 40% when compared to the healthy German norm population (control group). This SF-36 subscale is only one out of three subscales that provide information about the overall physical health. In this study, the other two subscales were found to be approximately 86% (subscale physical functioning) and 95% (subscale bodily pain) of the values found in the German norm population.

Although the postoperative quality-of-life scores after microvascular reconstruction were found to be lower than in the healthy control group, all mean subscales except for the role physical and general health were in the upper third (more than 66%) of the healthy German norm population.

Despite the quality of life and the donor-site morbidity assessment being performed using established standard methods, some limitations must be considered when interpreting the results of this study.

Firstly, the study’s sample size could potentially reduce the comparability of the used reconstruction technique with other methods and kind of prohibits generalising the research results. However, in the literature, many reports dealing with the assessment of quality of life and donor-site morbidity of different microvascular reconstruction methods investigated a similar patient size [1,5,6,18,36]. Secondly, in this study, no further investigation was performed concerning the quality of life and donor-site morbidity between radiated patients and patients who did not receive radiation therapy. Therefore, the influencing effect that results from radiation therapy on the patient’s quality of life could not have been evaluated in this study. Thirdly, no quality of life or upper limb disability assessment was conducted preoperatively for a direct comparison of pre- and postoperative values. However, most previous reports about donor site morbidity and quality of life after microvascular free flap reconstruction also did not include preoperative assessments due to their study designs [5,6,26].

## 5. Conclusions

The present study evaluates the TDAP-Scap-aa free flap regarding the postoperative donor site morbidity, the quality of life and the clinical data within a homogenous patient collective. This work demonstrates a mild donor-site morbidity of the scapular region approximately one year after flap harvesting. The overall quality of life was found to be in the upper third compared to the German norm population.

The donor-site morbidity and the quality-of-life outcome in this study suggest that the TDAP-Scap-aa flap might be a functional beneficial method when complex maxillofacial soft and hard tissue defects are reconstructed by using only one flap. As a future work project, further studies on a prospective and/or a multicentre basis with large patient collectives and a baseline assessment (DASH, SF-36 scores) are required to analyse the TDAP-Scap-aa free flap technique in more detail.

## Figures and Tables

**Table 1:** Case characteristics and postoperative individual DASH scores.

Case Characteristics				Tumour Classification ^1^			Defect Classification ^2^	DASH Score (Mean)
Case No.	Age (Years)	Sex (m/f)	Diagnosis	T	N	M		
1	64	M	HNSCC	4	2	0	IIB2	23.3
2	43	M	HNSCC	4	2	0	IB	28.3
3	61	M	HNSCC	3	1	0	IIB2	20.8
4	68	M	HNSCC	3	0	0	*IIB*	35.8
5	68	F	HNSCC	4	0	0	*IIIB*	20
6	56	M	ORN	-	-	-	IIC	32.5
7	43	M	HNSCC	4	1	0	IB	20.8
8	63	F	HNSCC	2	1	0	IIIB	22.5
9	69	M	ORN	-	-	-	IIIB	25.8
10	52	M	ORN	-	-	-	IC	35
11	77	F	HNSCC	4	1	0	IIIB	9.2
12	47	M	HNSCC	4	2	0	IIB1	10.8
13	53	M	ORN	-	-	-	ID	16,7
14	62	M	ORN	-	-	-	IIID	24.2
15	67	M	HNSCC	4	2	0	IIB2	14.2
16	75	F	HNSCC	3	2	0	IIIB	23.3
17	36	F	ORN	-	-	-	IIC	19.2
18	64	M	HNSCC	2	1	0	IID	21.7
19	74	M	HNSCC	4	0	0	IIB2	15.8
20	58	M	HNSCC	4	2	0	IIB2	15

^1^ Pathological TNM; ^2^ The mandibular defects were classified using Cordeiro’s mandibular defect classification [16], whereas maxillary defects (indicated by italic script) were classified according to Brown’s classification of maxillectomy and midface defects [17]. DASH = Disabilities of the Arm, Shoulder and Hand questionnaire.

**Table 2:** Summary data of the SF-36 quality-of-life results.

	Min	Max	Mean	SD	German Population (Mean)	Significance (*p*)
Physical Functioning (PF)	40	95	73.25	16.08	85.71	*p* = 0.003
Role Physical (RP)	0	75	33.75	23.33	83.7	*p* < 0.001
Role Emotional (RE)	0	100	78.34	31.12	90.35	*p* = 0.102
Vitality (VT)	30	70	43.25	10.04	63.27	*p* < 0.001
Mental Health (MH)	32	96	56.60	14.47	73.88	*p* < 0.001
Social Functioning (SF)	38	100	60.62	17.34	88.76	*p* < 0.001
Bodily Pain (BP)	45	100	74.25	15.90	79.08	*p* = 0.188
General Health (GH)	15	80	42.75	12.92	68.05	*p* < 0.001
Health Change (HC)	25	100	70.00	19.19		

Minimum, maximum, standard deviation and mean values are given in SF-36 questionnaire points.

## Data Availability

The data presented in this study are available on request from the corresponding author. The data are not publicly available due to privacy restrictions.
